# Memory B Cells in Multiple Sclerosis: Emerging Players in Disease Pathogenesis

**DOI:** 10.3389/fimmu.2021.676686

**Published:** 2021-06-08

**Authors:** Krista D. DiSano, Francesca Gilli, Andrew R. Pachner

**Affiliations:** Department of Neurology, Geisel School of Medicine & Dartmouth-Hitchcock Medical Center, Lebanon, NH, United States

**Keywords:** memory B cells, multiple sclerosis, neuroinflammation, B cells, multiple sclerosis-drug therapy

## Abstract

Multiple Sclerosis (MS) is an inflammatory demyelinating disease of the central nervous system. Once thought to be primarily driven by T cells, B cells are emerging as central players in MS immunopathogenesis. Interest in multiple B cell phenotypes in MS expanded following the efficacy of B cell-depleting agents targeting CD20 in relapsing-remitting MS and inflammatory primary progressive MS patients. Interestingly, these therapies primarily target non-antibody secreting cells. Emerging studies seek to explore B cell functions beyond antibody-mediated roles, including cytokine production, antigen presentation, and ectopic follicle-like aggregate formation. Importantly, memory B cells (Bmem) are rising as a key B cell phenotype to investigate in MS due to their antigen-experience, increased lifespan, and rapid response to stimulation. Bmem display diverse effector functions including cytokine production, antigen presentation, and serving as antigen-experienced precursors to antibody-secreting cells. In this review, we explore the cellular and molecular processes involved in Bmem development, Bmem phenotypes, and effector functions. We then examine how these concepts may be applied to the potential role(s) of Bmem in MS pathogenesis. We investigate Bmem both within the periphery and inside the CNS compartment, focusing on Bmem phenotypes and proposed functions in MS and its animal models. Finally, we review how current immunomodulatory therapies, including B cell-directed therapies and other immunomodulatory therapies, modify Bmem and how this knowledge may be harnessed to direct therapeutic strategies in MS.

## Introduction

Multiple Sclerosis (MS) is a chronic inflammatory demyelinating disease of the central nervous system (CNS), with a highly variable and unpredictable disease course that can manifest as a variety of physical and cognitive symptoms. Although cellular inflammation in MS has historically focused on one key player in adaptive immunity, T cells, B cells are now recognized as central mediators in MS pathogenesis. B cell antibody-mediated immunity has been implicated in MS pathogenesis since the discovery of elevated CSF IgG in 1942 (1). Subsequently, in 1959 oligoclonal bands (OCBs) in the cerebrospinal fluid (CSF) were identified (2) and, to date, OCBs remain a diagnostic hallmark in MS (3). OCB presence indicates niches of clonally-related antibody-secreting cells (ASC), including plasmablasts and plasma cells, within the CNS. Since the discovery of OCBs in MS, researchers have dedicated intense focus towards identifying the antigenic targets of ASC in the CNS compartment. However, in contrast to CNS neuroinflammatory diseases such as neuromyelitis optica, with clear autoantibody targets (aquaporin-4), probing antibody specificity in MS has not revealed consistent targets (4, 5), with some studies implicating diverse CNS self-antigens (6, 7) and viral antigens (8). The role of ASCs and OCBs in MS still remains elusive, with suggested involvement in pro-inflammatory functions, including autoantibody production, antibody- or complement-dependent cellular cytotoxicity, and opsonization, or anti-inflammatory functions, including production of the anti-inflammatory cytokine IL-10 (9, 10).

Further interest in the role of non-ASC B cells as key players in the MS immunopathogenesis followed the relatively recent success of B cell depletion therapies targeting CD20. These therapies, including rituximab (11, 12), ocrelizumab (13), and ofatumumab (14) reduced new inflammatory lesions and relapses despite the sparing of most ASCs, i.e. CD20^-^ plasma cells and some plasmablasts. These novel findings fueled considerable interest in examining the phenotype and function of non-ASC B cells in MS. Current research seeks to explore B cell function in MS beyond antibody-dependent roles to define antibody-independent mechanisms, including antigen presentation, cytokine production, and ectopic lymphoid follicle-like structures. Among non-ASC B cell subtypes, increased attention has been directed towards the role of memory B cells (Bmem) in regulating immune processes in MS. Bmem have several unique features, including increased longevity, the capacity to rapidly respond to re-exposure to antigen, and the ability to serve as direct antigen-experienced precursors to antibody-secreting cells. Due to the relatively recent interest in Bmem, our knowledge regarding the exact functions of Bmem in MS is expanding. This review aims to explore our current understanding of this key component of immunological memory in MS and its animal models.

In the first part of this review, we summarize the current knowledge regarding Bmem development, trafficking, phenotypes, and function during homeostasis and inflammatory conditions, providing a basis for understanding the mechanisms in which Bmem may contribute to MS and are targeted by immunomodulatory therapies.

In the second part of this review, we describe Bmem in MS and its animal models reviewing phenotypes and putative functions, and finally, we examine the effectiveness of current therapeutic approaches in targeting Bmem.

## Bmem Development

A key player in immunological memory, Bmem can be defined as a B cell that has encountered antigen and remains in a quiescent state until re-exposed to antigen, at which point the cell rapidly responds to the second challenge. Upon first pathogen encounter, the majority of Bmem are derived from germinal center (GC) reactions. GCs are specialized structures within secondary lymphoid tissue (SLT) where mature, antigen-experienced B cells undergo cognate interactions with T cells, proliferate, undergo somatic hypermutation to increase B cell receptor (BCR) affinity for antigen, perform immunoglobulin (Ig) isotype switching, and are selected based on affinity for a specific antigenic target. Select GC B cells ultimately differentiate to produce antigen-specific, isotype-switched ASC or Bmem. Though GC B cells serve as the precursor for both ASC and Bmem, the mechanisms regulating Bmem versus ASC differentiation remain poorly understood. Numerous factors have been proposed to contribute to Bmem formation, but no “master regulator” for Bmem differentiation has been identified. Animal models have suggested the transcription factor BACH2 selects GC B cells with intermediate affinity to differentiate into Bmem (15). Additionally, Bmem generation is associated with an increased expression of factors including ZBTB32 (16), KLF2 (17, 18), ABF-1 (19), STAT5, BCL-6 (20, 21), and SKI (21), which, in general, repress differentiation to an ASC phenotype. Cytokines, including IL-24 (22) and IL-9 (17) can enhance Bmem formation. Moreover, *in vitro*, IL-2, IL-10, and CD40L were demonstrated to be involved in differentiating GC B cells to a Bmem phenotype (23). Outside of GC, a small proportion of antigen-experienced B cells may additionally be selected for based on low affinity to form Bmem in an early wave prior to GC formation (24, 25). GC-independent isotype-unswitched (IgM) or –switched (IgG) Bmem exhibit low affinity due to unmutated Ig variable genes (26). In humans, few Bmem lack somatic mutations for antigen (27), suggesting most Bmem are GC-derived. Following Bmem formation, these cells may reside in survival niches including SLT such as the spleen (28) for years in a resting state independent of antigen; however, these niches are localized near areas of antigen encounter (29). Bmem are also observed in the tonsils and the bone marrow and may enter into circulation to patrol at low levels (28). Bmem express higher levels of the adhesion molecules LFA-1 and VLA-4 compared to naive B cells, with VLA-4 primarily mediating Bmem retention in SLT (30). *In vitro*, Bmem migrate towards CXCL12 (23, 31), CCL19, and CXCL13 (23, 32) suggesting these chemokines may be involved in movement within the SLT and trafficking to survival niches or sites of inflammation. If the humoral immunity generated from long-lived plasma cells residing in the bone marrow is not sufficient to eliminate pathogens, Bmem become actively involved in the inflammatory response. Upon re-exposure to antigen, Bmem will generate a more rapid and potent antigen-specific response relative to naïve B cells (33).

## Bmem Phenotypes

**Figure 1:**
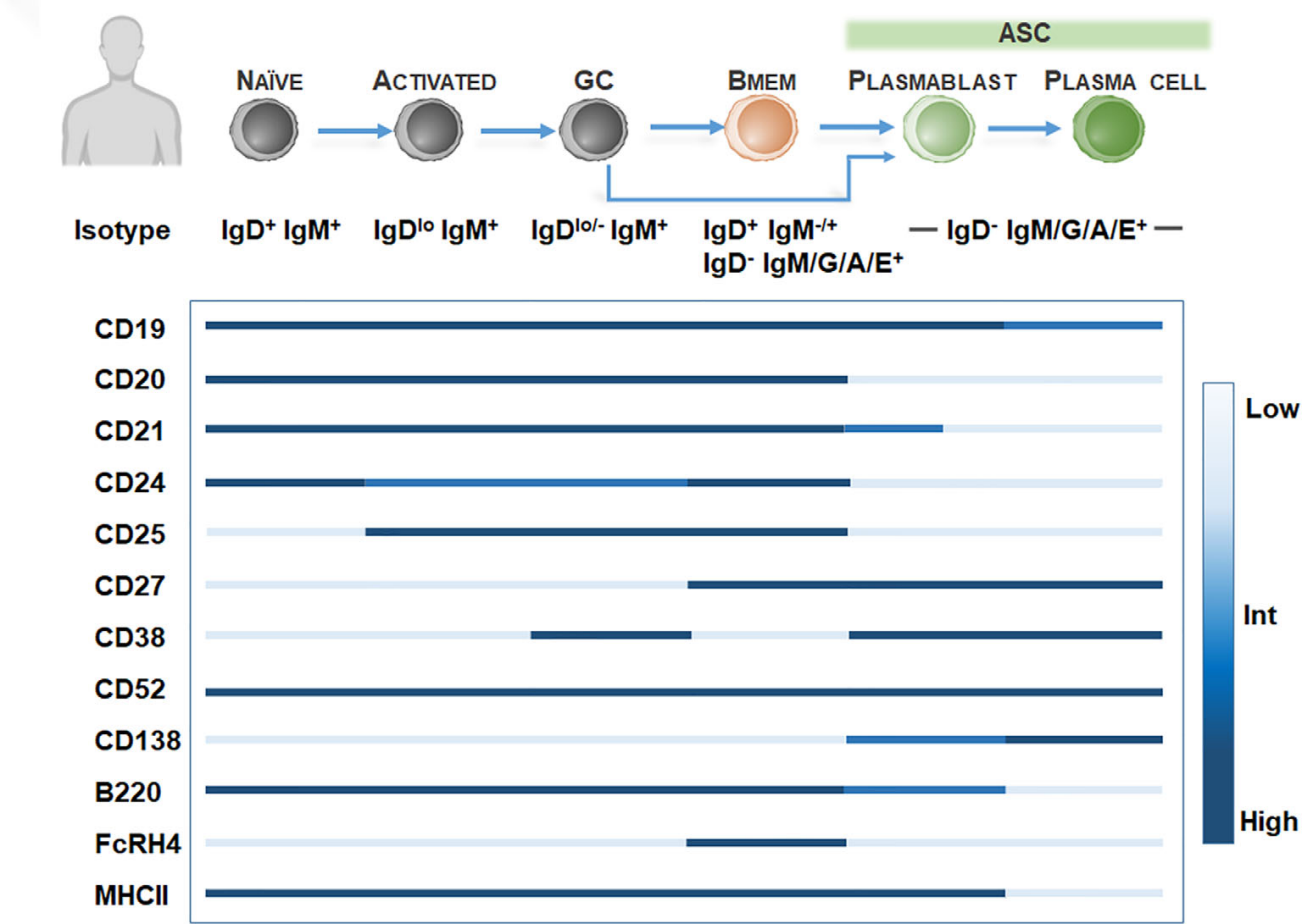
Bmem phenotyping markers in humans. Overview of B cell differentiation in humans, focusing on naïve B cell to ASC phenotypes, with BCR isotype and surface marker expression. Surface marker levels simplified to highlight relative expression, including low, intermediate, or high levels.

In humans, Bmem are conventionally identified by the expression of tumor necrosis factor superfamily member CD27, a protein regulating entry into plasma cell lineage and properties associated with Bmem including isotype switching and Ig variable gene mutation (34, 35). However, CD27 is not exclusive to Bmem and is likely a marker of GC and post-GC activation as CD27 is also expressed on GC B cells and post-GC B cells, including ASC (**Figure 1**). Thus, CD27 expression should be coupled with low levels of CD23 (36) and the lack of expression of the ASC marker CD138 (syndecan-1) to identify Bmem in humans. Further inclusion of specific patterns of CD38 (37), CD21 (38), CD24 (39), CD19 (40), B220 (41), FCRL4 (FcRH4) (38, 42) and CD25 (43, 44) can delineate heterogeneous Bmem populations (**Figure 1**). Thus far, the main populations of CD19^+^CD27^+^CD138^-^ Bmem present in peripheral blood and bone marrow include three isotype-unswitched Bmem phenotypes, including IgM^+^IgD^+^, IgM^-^ IgD^+^, IgM^+^IgD^−^ (IgM-only memory cells), and isotype-switched IgM-IgD- phenotypes, including IgG, IgA, or IgE+ Bmem. Bmem are typically isotype-switched and primarily express IgG subclasses. IgG+ Bmem comprise 15-20% of peripheral blood B cells, including predominately IgG_1_, IgG_2_, IgG_3_ subclasses (45). Among IgG Bmem, it should be noted that a small proportion of “atypical” IgG Bmem may lack CD27 (38, 45, 46). Isotype-switched IgA Bmem comprise around 10% of B cells in peripheral blood and are generally implicated in mucosa-associated tissues (45) while IgE Bmem involved in allergic responses are rarely detectable in humans and mice and their development and lifespan is poorly understood (45). Among isotype-unswitched phenotypes, IgM and IgD-expressing Bmem, including IgM^+^ IgD^+^ (15% of B cells), IgM^-^ IgD^+^ (1%), or IgM^+^IgD^-^ (5%) may be found within the blood or bone marrow (34, 47, 48).

**Figure 2:**
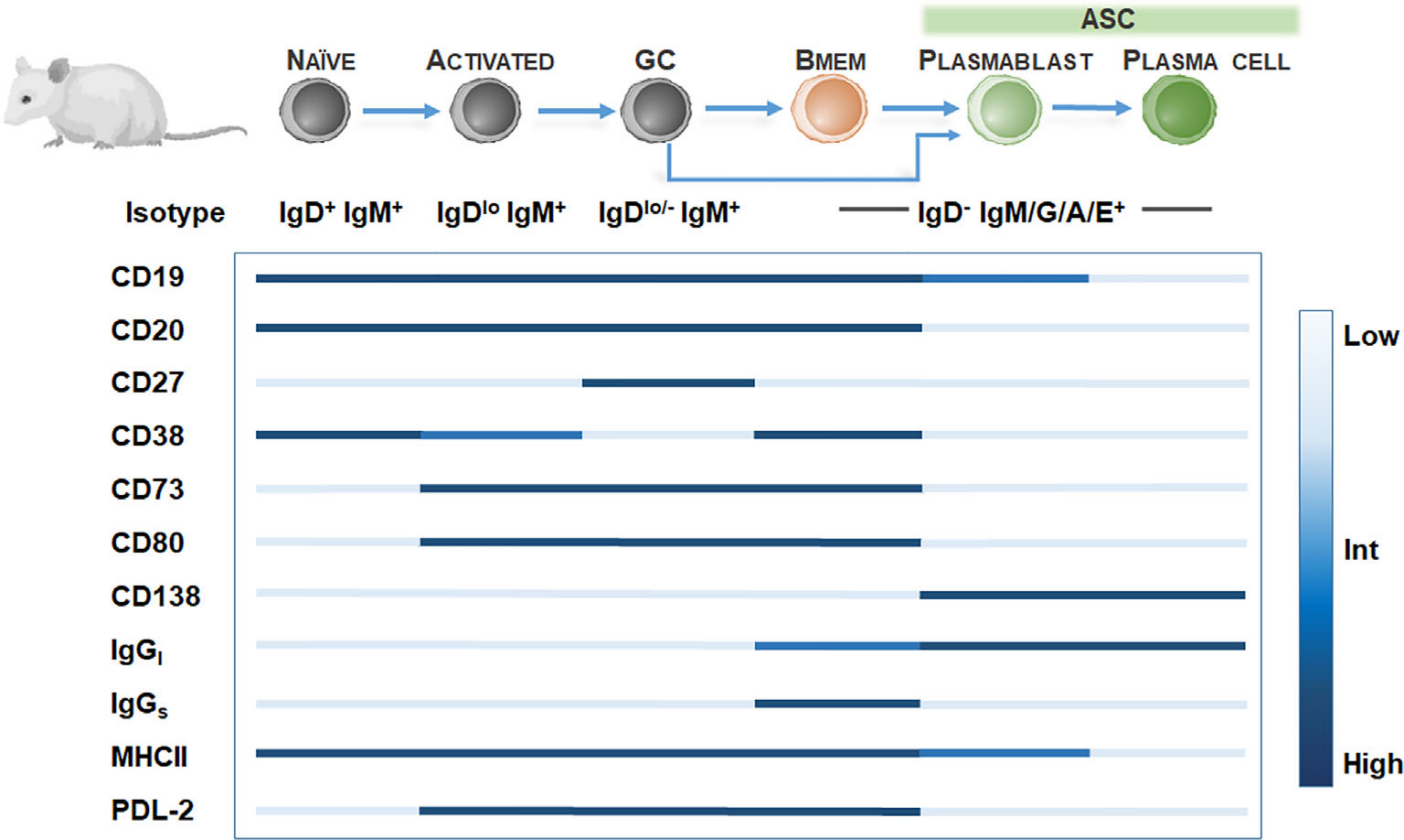
Bmem phenotyping markers in murine models. Overview of B cell differentiation in mice, focusing on naïve B cell to ASC phenotypes, with BCR isotype and surface marker expression. Surface marker levels simplified to highlight relative expression, including low, intermediate, or high levels.

In rodent models, Bmem identification is hampered by the low frequency of Bmem (49) and the lack of CD27 expression among Bmem (50). Further definitive Bmem markers in mice have remained elusive. Exploration of novel Bmem markers in mice have relied on several methods including 1) boosting Bmem frequencies using antigen-based cell enrichment protocols (51, 52), 2) protein immunization in BCR transgenic mice with a fixed BCR specificity (29), 3) adoptive transfer of antigen-specific B cells (53), or 4) genetic tagging of activation-induced cytidine deaminase (AID), an enzyme essential for isotype switching and somatic hypermutation identifying GC-derived B cells including Bmem and ASC (33). Murine studies have proposed at least 10 Bmem subsets utilizing Ig isotyping combined with surface expression of CD80 (49, 54, 55), PDL2 (54, 55), CD73 (55, 56), CD38 (57). However, these markers may be expressed on other murine B cell subtypes, so a diverse panel of surface markers is necessary for identifying Bmem (**Figure 2**). For isotype-switched Bmem, IgG surface (IgGs) versus intracellular (IgGi) expression (58, 59) in combination with CD138 or Blimp-1 (60, 61) may be used to distinguish ASC (IgGi^hi/+^, igGs^low^, CD138^+^, Blimp-1^+^) (62) and Bmem (IgGi^low^, IgGs^hi/+^, CD138^-^, Blimp-1^-^). Moreover, similar to assaying human Bmem, *in vitro* stimulation using polyclonal activators (i.e. CpG DNA, R848 TLR7/8 agonist) to convert Bmem into ASC, combined with a conventional Enzyme-linked ImmunoSPOT (ELISPOT) assay, may be used to quantify Bmem and determine antigen specificity and Ig isotype in mice (63–65).

## Bmem Function

Compared to naïve mature B cells, Bmem display several distinctive features. Bmem have enhanced longevity and can survive for years and perhaps for the lifetime of the host independent of antigen (66, 67). In comparison, naïve mature B cells have a lifespan of weeks (68). Furthermore, since most Bmem are GC-derived, Bmem are generally clonally expanded, isotype-switched, and have undergone somatic hypermutation of Ig variable genes to increase antigen affinity. Unlike naïve or activated mature B cells, Bmem are able to rapidly proliferate and differentiate into ASC with minimal stimulation requirements, including re-exposure to low levels of antigen (69, 70), T cell help (71–73), or polyclonal stimulation (73, 74). Bmem enter cell cycle, differentiate into ASC, and potentially re-seed GC quicker than mature B cells (75, 76). These advantages are likely due to a combination of factors, including reduced quiescence factors (Kruppel-like factor 4 and 9; PLZF) (77), higher expression of co-stimulatory molecules (CD80, CD86) (78, 79), CD27 (50), IL21R (80), SLAM (signaling lymphocytic activation molecule) (79), TLR7/9 (81), and anti-apoptotic molecules (BCL2) (82). Once activated, Bmem can follow two paths: 1) rapidly differentiating into ASC or 2) re-entering into secondary GC reactions to undergo further affinity maturation and isotype-switching. In murine studies, IgG Bmem show a greater proclivity to differentiate into ASC, while IgM Bmem are often selected for re-entry in GC reactions (33, 51). Bmem differentiating to ASC can contribute to the rapid and copious production of high affinity antibodies to supplement antibody produced by terminally differentiated plasma cells residing in niches, such as the bone marrow. In addition to rapid differentiation to ASC, Bmem are potent antigen-presenting cells (APCs), expressing MHCII (83) that enables not only the efficient recognition of antigen, but the ability to process antigen for presentation to activate other immune cells, including T cells (84). Finally, Bmem produce a wide array of cytokines including TNF (85, 86), GM-CSF (86), IL-6 (86, 87), lymphotoxin (LT) (85), and IL-10 (85).

## Bmem in Multiple Sclerosis

In MS, B cells are located within multiple compartments in the CNS, including the CSF, parenchyma, and meninges. However, studies exploring Bmem in MS have primarily focused on the peripheral blood and CSF, with few studies examining Bmem localization in the parenchyma and meninges. Among these studies, there are notable discrepancies in defining Bmem, with the majority of studies defining Bmem based exclusively on CD27 expression. Therefore, for each mentioned study, the surface markers utilized to define Bmem will be noted.

### Phenotype, Trafficking, and Localization

In MS, Bmem frequencies are elevated in the CSF compared to peripheral blood (88, 89) and Bmem comprise the majority of B cells populating the CSF (90, 91) (CD27^+^ IgD^-^ (88, 91); CD19+CD27+ (89); CD27+ CD138- (90); CD19+ CD27+ IgD- and IgD+). In contrast to the peripheral blood, the proportion of CD19^+^ B cells among total lymphocytes is significantly lower in the CSF (91). However, the proportion of class-switched B cells, including isotype-switched Bmem, among CD19+ B cells is enriched in the CSF (91). Further studies have confirmed the majority of Bmem populating the CSF display an isotype-switched phenotype (71%; CD19^+^ CD27^+^ IgD^-^ IgM^-^) (92). In agreement with these findings, B cells populating the CSF, including Bmem, bear extensive somatic mutations and exhibit clonal expansion (88). Conversely, in a recent pre-print, Bmem in peripheral blood from MS patients displayed an Ig isotype distribution of 50% IgM, 30% IgA, and 20% IgG (93). In MS patients, ASC populating the CSF exhibit a selective enrichment towards the IgG1 allotype G1m1 compared to the peripheral blood (94). In a recent pre-print, Bmem in the intrathecal compartment did not exhibit the same dominance towards the G1m1 allotype constant region polymorphism, suggesting certain B cell-lineages may preferentially differentiate (95). To date, it remains unclear if skewed Ig allotypes influence MS risk and phenotype (96, 97).

Bmem are not restricted to the CSF compartment, and Bmem (CD27^+^) are found within the brain parenchyma (98, 99). Furthermore, B cells recovered from MS plaques display mutations and clonal expansion (100, 101), suggesting primarily differentiated B cells (Bmem/ASC) occupy the parenchymal space, similar to the CSF. It has been suggested that BCR mutations and clonal expansion may be acquired in the CNS compartment (89), possibly aided by inflammatory aggregates in the brain meninges mimicking some features of ectopic lymphoid follicles (102). In a recent pre-print, extensive clonal connections were found among Bmem and ASC in the CSF compartment (95). Clonal connections between Bmem and ASC were also found to span different isotypes, including IgM/IgG1, IgG1/IgG2, and IgM/IgA1. These findings suggest ASC and Bmem share a common origin, although it remains unclear whether these clonal similarities originate in the periphery or the intrathecal compartment. At least a proportion of B cells appear to undergo an active exchange between the periphery and CNS in MS, with CD27^+^ IgD- B cells sharing similar repertoires between the peripheral blood and CSF (91, 103). Moreover, Stern et al. demonstrated the B cell clonal families observed in MS brain tissue were frequently derived from founders in the deep cervical lymph nodes (104). Regardless of the mechanism promoting Bmem persistence in the CNS, the exact chemokines initiating and/or sustaining Bmem trafficking to the CNS compartment in MS remain to be determined. Several chemokine receptors including CXCR4 (105), CXCR5 (91), CXCR3 (95), CCR1, CCR2 and CCR4 (88) have been implicated in trafficking and are upregulated on CSF B cells compared to paired-peripheral blood. Adhesion molecules regulating Bmem entry into the CNS meninges and parenchymal compartments are less clearly understood. VLA-4 has been implicated in aiding B cell transmigration in ex vivo culture studies (106) and murine studies (107), though these studies have examined global B cell migration and further studies are required to determine whether VLA-4 is essential for Bmem transmigration.

### Function

#### Antibody Production and Antigen Specificity

Tracking Bmem conversion into ASC to investigate antibody production and specificity *in vivo* remains challenging and often requires specialized murine models. Alternatively, *in vitro*, Bmem can be stimulated to convert into ASC utilizing polyclonal activators specifically triggering Bmem differentiation, including the TLR7/8 agonist R848 (108, 109). Bmem may subsequently be quantified and Ig isotype and antibody production may be evaluated. Limited studies exist examining Bmem conversion to ASC and antibody production in MS. Hohmann et al. isolated B cells from the peripheral blood of MS patients and compared IgG antibodies produced by ASCs or Bmem-derived ASCs, i.e. B cells *in vitro* stimulated using R848 and IL-2 by ELISPOT (110). Bmem-derived ASCs generated larger spot size compared to ASCs, suggesting enhanced IgG secretion from Bmem-derived ASCs.

B cell antigen specificity in MS has remained unclear and is documented as heterogeneous, with antibody targets ranging from self-antigens to viral antigens. With regards to Bmem, there have been few studies on this topic. Hohmann et al. exclusively examined reactivity to normal human brain lysates (110). Among 15 of the 30 relapsing-remitting MS (RRMS) patients tested, brain-reactive Bmem-derived ASC were present in the peripheral blood. In some patients, brain-reactive Bmem were present in relapse and remission, while other patients displayed brain-reactive Bmem in the relapse only. The presence of brain-reactive B cells, including Bmem, predicted relapse. Brain-reactive B cells were not observed in the peripheral blood of healthy donors or other neurological disease controls (111).

#### Antigen Presentation

Bmem are conventionally regarded as potent APCs. In MS, CSF Bmem (CD27^+^ IgD^-^) display upregulated expression of two co-stimulatory molecules key in antigen presenting functions, CD80 and CD86, compared to naïve B cells (88). Although this is a well-known feature of Bmem regardless of disease pathogenesis, this finding suggests Bmem in the CSF of MS patients also display an enhanced ability to engage with immune cells, including T cells. In alignment with these findings, *ex vivo* Bmem (CD19^+^ CD27^+^) isolated from RRMS patients elicited autologous CD4 T cell proliferation in the presence of antigens including, tetanus toxoid, myelin basic protein (MBP), and myelin oligodendrocyte protein (MOG) (112). Moreover, Bmem isolated from some RRMS patients are capable of activating CD4 T helper (Th) cells in the presence of myelin antigens *in vitro*, inducing T cell proliferation and IFNγ production (112). Furthermore, the *in vitro* spontaneous proliferation of Th1 cells observed in patients carrying the risk allele HLA-DR15 was found to be mediated by Bmem (CD27^+^) with high MHCII surface receptor HLA-DR expression (113).

#### Cytokine Production

B cells, including Bmem, in MS patients may exhibit a propensity towards a dysregulated cytokine network. An increased frequency of Bmem (CD27^+^) producing GM-CSF was observed in the peripheral blood obtained from MS patients compared to healthy controls (86). Furthermore, *in vitro* stimulated B cells isolated from the peripheral blood of RRMS and SPMS patients exhibit a decreased production of the anti-inflammatory cytokine IL-10 compared to healthy controls, while LT and TNF levels were comparable (85). Further studies demonstrated stimulated Bmem (CD19^+^ CD27^+^) obtained from RRMS patients produce elevated LT and lower IL-10 than naïve B cells (112). However, Bmem isolated from healthy donors produced comparable levels of both cytokines. *In vitro* stimulated Bmem obtained from healthy donors also exhibited lower levels of IL-10 production compared to naïve B cells (85), thus, low levels of IL-10 production seems to be typical Bmem feature regardless of disease pathogenesis. The reduced IL-10 production by B cells observed in RRMS and SPMS patients may therefore be attributed to another B cell phenotype, including IL-10-producing regulatory B cells or ASC (9, 114).

#### Associations With Clinical Disease

Recent studies have sought to investigate the association of Bmem with clinical outcomes in MS. In RRMS patients, an increased CD5^+^ Bmem subpopulation was associated with the remitting stage compared to the relapsing stage (115). Furthermore, Nissimov et al. demonstrated elevated peripheral blood Bmem frequencies were associated with a lower expanded disability status scale score (116). Conversely, Comabella et al. determined that increases in isotype-unswitched and -switched Bmem (CD19^+^ CD27^+^ IgD^+^ or IgD^-^) in the peripheral blood from RRMS patients were associated with an MRI phenotype with high neurodegeneration, defined by increased contrast-enhancing lesions and non-enhancing black holes on T1-weighted images, and decreased brain parenchymal fraction (117). Bmem populations also differ in peripheral blood obtained from pediatric and adult MS patients (118). In pediatric MS, Bmem (CD20^+^ CD27^+^) are elevated in the peripheral blood compared to healthy children and adolescents. In contrast to adult MS patients who display elevated isotype-switched Bmem (CD20^+^ CD27^+^ IgD^-^) and plasma cells in peripheral blood, non-switched Bmem (CD20^+^ CD27^+^ IgD^+^) and plasmablasts were increased in frequency in pediatric MS patients.

## Bmem in Animal Models of MS

Murine models of MS generally have been limited in exploring Bmem due to the lack of conventional Bmem markers, the low quantity of Bmem (25), the shifted surface expression of proposed murine markers on Bmem isolated from CNS compartment (63), and the time-consuming methods utilized to isolate Bmem and quantify by *in vitro* stimulation assays (64, 65). In this section, we will review data on Bmem obtained from pre-clinical models of MS, including two viral models of demyelination, mouse hepatitis virus (MHV) and Theiler’s murine encephalomyelitis virus (TMEV), and the autoimmune model, experimental autoimmune encephalomyelitis (EAE).

### Viral Models of Demyelination

Viral immune-mediated demyelination models emulating features of MS, including MHV (coronavirus family) and TMEV (picornavirus family), require B cell and antibody responses for viral control (119, 120) and recruit diverse B cell subtypes CNS (59, 121). There is also evidence for B cell involvement in demyelination and clinical disability (122–125).

Intracerebral MHV infection, including the A59 and JHM strains, induces an acute inflammatory demyelinating disease, with prominent B cell CNS infiltration mimicking the acute inflammatory stages of MS. In MHV models, Bmem are present in the CNS parenchyma as evaluated by flow cytometry (59), genetic tagging of AID-expressing B cells (126), and *in vitro* stimulation and evaluation *via* ELISPOT assays (63). Among total CNS-infiltrating Bmem (CD19^+^, CD138^-^, IgD^-^, IgG2a/b surface+, IgG2a/b intracellular^low^) the majority comprise an IgG2a/2b isotype-switched phenotype. ELISPOT analysis of *in vitro* stimulated Bmem determined that ASC and Bmem are initially recruited to the CNS (brain/spinal cord) with similar kinetics, but during the chronic phase of infection (day 35 post infection-p.i.), virus-specific IgG ASC persisted at higher frequencies than IgG Bmem in the spinal cord, the predominant site of inflammation and demyelination (63). ELISPOT analyses revealed that antibody production levels were similar between ASC and Bmem-derived ASC in both brain and spinal cord tissues. Gene expression analysis of chemokine receptors on CNS-infiltrating Bmem (CD19^+^ IgD^-^ CD138^-^) revealed highly upregulated expression of CXCR3 and CCR7, with moderate expression of CXCR4 and CXCR5 (59). Compared to ASC (CD138^+^), Bmem expressed higher levels of CCR7 and CXCR5, with similar expression of CXCR4, and lower expression of CXCR3. These results suggest multiple chemokine receptors may be simultaneously regulated on Bmem to direct recruitment. AID-genetically tagged Bmem and ASC were continually recruited from the periphery to the CNS concurrent with GC maturation (126). Moreover, once recruited to the CNS, there was no evidence of AID mRNA expression among Bmem, suggesting these cells were not undergoing somatic hypermutation or isotype switching in the CNS compartment during chronic infection (59). It still remains unclear whether Bmem are required for sustaining the local antibody production responsible for controlling viral recrudescence. Future studies are also required to determine if Bmem contribute to antibody-independent functions, including local cytokine production and antigen presentation.

In the chronic progressive demyelinating disease model, TMEV-induced demyelinating disease (TMEV-IDD), intracranial infection with TMEV mimics several neurodegenerative and clinical features of progressive MS (127). In chronic disease (day 120 p.i.) a phase of accumulating disability, Bmem (IgG^+^ CD138^-^) were identified in spinal cord tissue (121). Although the function of Bmem in TMEV-IDD remains to be determined, B cell depletion therapy (anti-CD20) targeting non-ASC B cells, including Bmem, exacerbated microglial activation, increased T cell infiltration, demyelination, and axonal damage (123).

### Autoimmune Models

Although a wide array of EAE models exist, the most commonly utilized EAE models emulate the acute or relapsing/remitting stages of MS (128) and are induced independent of B cells (128–130). Due to the limited B cell involvement in these models, including the MOG_35-55_ peptide model induced in C57BL/6 mice, the role of Bmem in EAE models remains relatively unexplored.

Several therapeutic interventions targeting B cell subtypes including Bmem may provide insights into Bmem function in EAE autoimmune models of MS. In anti-CD20 studies in EAE, clinical disease is suppressed in murine MOG_35-55_ (131, 132) and marmoset EAE models (133, 134). CD20 depletion was also found to ablate IL-6 producing B cells (131), including Bmem. In a T-independent protein immunization murine model (TNP-LPS) anti-CD20 administration depleted existing and adoptively transferred Bmem (135). Mice deficient in B cell maturation antigen (BCMA), an important receptor for B cell-activating factor (BAFF) and a proliferating inducing ligand (APRIL) regulating ASC differentiation and survival, showed exacerbated EAE disease severity (136). *In vitro*, BCMA expression directly inhibited Bmem expansion and anti-inflammatory cytokine production, suggesting BCMA deficient mice may show increased proportions of Bmem. Together, these studies suggest Bmem may contribute to EAE pathogenesis. However, other therapeutic interventions have suggested Bmem may play a dispensable or, perhaps, beneficial role in EAE pathogenesis. Atacicept, a TACI fusion protein that inhibits the B cell survival factors B lymphocyte stimulator (BlyS) and APRIL, spares B cell progenitors and Bmem (137). Atacicept’s use has been explored in both the B cell-dependent recombinant human MOG_1-125_ (rhMOG) and B cell-independent MOG_35-55_ models. In both models, prophylactic treatment resulted in reduced B cell infiltration into the CNS, delayed disease onset, and attenuated disease severity (138). In addition, a key cytokine promoting Bmem survival, IL-15, was found to be enhanced in a murine lupus model following TACI-IgG treatment (139).

Altogether, further studies are required to determine Bmem function in EAE models of MS as anti-CD20 therapies, atacicept, and BCMA deficiency all affect multiple B cell subsets. Following the success of B cell-depleting therapies in MS, increasingly studies are utilizing B cell-dependent EAE models, including rhMOG EAE and EAE induced in IgH^MOG^ transgenic mice where 30% of B cells are specific for MOG (140). Future studies utilizing these models may pinpoint the exact Bmem phenotypes and Bmem functions involved in autoimmune models of MS.

## MS Immunomodulatory Therapies and the Effect on Bmem

### B Cell-Directed Immunomodulatory Therapies

B cell depletion therapies targeting CD20, including rituximab, ocrelizumab, and ofatumumab, deplete all B cells except ASC and pro-B cells (141) (**Figure 1**; **Table 1**) and have shown significant efficacy in reducing clinical relapse rates and new lesion formation in RRMS patients (11, 196). Additionally, in young, inflammatory primary progressive MS (PPMS) patients, ocrelizumab has been shown to reduce clinical disease progression and brain atrophy (197). Following anti-CD20 therapies, B cells including Bmem are significantly decreased in the peripheral blood of MS patients (142, 146) (**Table 1**), with dramatic peripheral B cell depletion still evident by 6 months post-treatment. In rituximab-treated patients, a reduction in CSF B cells was also observed in RRMS patients (147, 148), while PPMS patients were only shown to exhibit a moderate reduction (149). In RRMS patients, rituximab treatment was shown to normalize the ratio of GM-CSF to IL-10 producing B cells in the peripheral blood (86). Eight-to-24 months post-treatment, reappearing peripheral blood B cells were strongly diminished in memory B cells (116).

**Table 1:** Immunomodulatory MS treatment effects on Bmem and B cell function.

MS treatment	Target	Target cells/pathways	Bmem phenotypic markers	Memory B cells in blood	B cells in CNS compartment	Effects on B cell function	Outcome	FDA approval/clinical trial phase
Immunomodulatory: B cell-directed								
Rituximab	Chimeric mAb Anti-CD20	-Expressed on all B cells, but terminally differentiated plasma cells (141) -Some T cells express CD20 (142, 143) -Greater CDC than ADCC (144)	CD19+, CD27+, IgD- (142) CD19+ (145),CD27+ (86)	Decreased (142, 146)	RRMS: CSF CD19+ B cells decreased (147, 148) PPMS: Moderate reduction in CSF B cells compared to PB (149)	RRMS: Ratio of GM-CSF to IL-10 producing B cells in PB normalized (86)	RRMS: patients: -Reductions in new brain MRI lesions -Reduced clinical relapse rates	Phase II
Ocrelizumab	Humanized IgG1 Anti-CD20	-Expressed on all B cells, but terminally differentiated plasma cells -Some T cells express CD20 (143) -Greater ADCC than CDC (144)	N/A	Decreased total CD19+ B cells (150)	Decreased CD19+ B cells (151)	N/A	RRMS: -reduced gd- enhancing lesions and new lesion formation -reduced clinical relapses PPMS: -clinical progression reduced -reduction whole brain atrophy and WM lesion volume	FDA approved: RRMS and PPMS
Ofatumumab	Fully humanized IgG1 Anti-CD20	Expressed on all B cells, but terminally differentiated plasma cells -Some T cells express CD20 (143) -Greater CDC than ADCC activity (144)	N/A	Decreased total CD19+ B cells (145)	N/A	N/A	RRMS: -reduction in number of new gd+ lesions	Phase 2b
Atacicept	Fully human recombinant TACI fusion protein	-Blocks mature B cells and plasma cell survival -Memory B cells spared (137)	Rheumatoid arthritis: CD19+, CD20+, CD27+, CD38− (152)	-Increase in Rheumatoid arthritis patients (152)	N/A	N/A	RRMS: -Annualized relapse rates increased compared to placebo -Similar gd-enhancing lesions	Phase II -Early termination
Tabalumab	Fully humanized IgG4 mAb anti-BAFF (membrane bound and soluble)	Blocks immature/transitional B cells, naïve/mature B cells and plasma cell survival (153, 154)	CD19+, CD27+, IgD- (154) CD19+, CD27+, IgD+ (154)	-Increase (154)	N/A	N/A	RRMS: -No reduction in gd-enhancing lesions	Phase II
Inebilizumab MEDI-551	Humanized IgG1 mAb Anti-CD19 -Afucosylated IgG Fc region enhances ADCC (155, 156)	Targets pro-B cells through memory B cells, plasmablasts, and some plasma cells (155, 156)	N/A	-Total CD20+ (156, 157), and PC gene phenotype reduced (157)	N/A	N/A	RRMS: -Reduction in new gd-enhancing lesions over 24 weeks	Phase I
BTK inhibitors: -Evobrutinib -Tolebrutinib -Fenebrutinib -Orelabrutinib -BIIB091	BTK binding mechanism (158): Evobrutinib: Covalent, irreversible (159) Tolebrutinib: Covalent, irreversible Fenebrutinib: Non-covalent, reversible Orelabrutinib: Covalent, irreversible BIIB091: Non-colavent, reversible	B cells, myeloid cells, and hematopoietic cell lineages (160) except for T cells, plasma cells, and NK cells (161)	Evobrutinib: CD19+ CD20+ IgD-CD27+ CD38- (162)	Evobrutinib: No reduction in peripheral blood Bmem over 48 weeks (162)	N/A	Evobrutinib: Reduced CXCR3+ Bmem migration across human brain endothelial cells *in vitro* (163)	Evobrutinib: RRMS -Reduced gd-enhancing lesions -No effect on annualized relapse rates or disability progression Tolebrutinib: RRMS Reduced new gad-enhancing lesions -Reduced new or enlarging T2 hypointense lesions	Evobrutinib: Phase 3 Tolebrutinib: Phase 3 Fenebrutinib: Phase 3 Orelabrutinib: Phase 2 BIIB091: Phase 1
Immunomodulatory								
IFN-β therapies	Binds to Interferon α/β receptor (IFNAR)	Widespread reduction in cellular and molecular pro-inflammatory mediators and an increase in anti-inflammatory mediators (164)	CD19+, CD27+, CD38-, IgM- IgD- (165); CD27+, IgD-; and CD27+, IgD+ (166)	Decreased (165) Decreased (166)	N/A	-Decreased MHCII on B cells (167) -Reduced CD80+ (168) and CD40+ (169) B cells -Increased IL-10 production by *in vitro* stimulated B cells (168, 170)	RRMS: -Reduced relapses -Reduced MRI lesion activity -Reduced brain atrophy -Increased time to reach CDMS -Reduced risk of sustained disability progression	FDA approved: RRMS
Glatiramer acetate	Synthetic polypeptide mixture resembling myelin basic protein	Widespread effects on innate and adaptive immunity; suppression of pro-inflammatory mediators; increase in anti-inflammatory mediators (171)	CD27+, IgD- and CD27+, IgD+ (172)	Decreased (172)	(148)	-Reduced CD69, CD25, CD95 expression; decreased TNFα production; increased IL-10 production (173)	RRMS -Reduced relapses -Increased proportion of relapse free patients -reduction in gd-enhancing lesions and new lesions	FDA approved: RRMS
Cladribine	Synthetic chlorinated deoxyadenosine analog	Preferential depletion of T and B lymphocytes (174)	CD19+, CD27+, IgD-, IgM (175)	Decreased (175)	N/A	N/A	RRMS: -Reduced clinical relapse -increased proportion of relapse-free patients -increased proportion patients free from 3 month confirmed disability progression -reduced gd-enhancing lesions and active T2 lesions	FDA approved: RRMS
Fingolimod	Structural analog to sphingosine	S1P receptor expressing lymphocytes	CD19+, IgD+, CD27+; CD19+, IgD-, CD27+; CD19+, CD20+, CD27+ (93, 176) CD19+, CD27+, CD38int/high (177) CD27var, CD38- (178)	Decreased (177, 178)	No change in CSF B cell percentage (93, 179)	Impaired CSF B cell clonal expansion (93) -Reduced activation of memory b cells (177)	RRMS: -Reduced number and volume of gd-enhancing lesions -Reduced new and enlarging T2 lesions -Reduced relapse rate -Increased percentage of relapse-free patients -Delayed disability progression	FDA approved: RRMS
Dimethyl fumarate	Fumaric acid ester	Widespread anti-inflammatory properties, including shift from Th1 to Th2 profile (180)	CD27+ (181, 182) CD27var, CD38- (178) CD27+, IgA or IgG+ class-switched Bmem; CD27+, IgM+ unswitched-Bmem (183)	Decreased (178, 181, 182) Class-switched and unswitched both reduced (183)	Decreased (184)	-Reduction in GM-CSF, TNF-alpha, IL-6 producing B cells (181, 183) -reducing phosphorylation of STAT5/6 and NFƘB in surviving B cells (183) -IL-10 production by B cells intact (182)	RRMS: -Number of gd+ lesions reduced -Reduced new or enlarging T2 lesions and new T1 hyopintensities -Improved annualized relapse rate -Reduced risk of disability progression	FDA approved: RRMS
Teriflunomide	Active metabolite of leflunomide	Rapidly proliferating cells, including T and B cells *via* inhibition of *de novo* pyrimidine synthesis (185)	CD19+, CD27dim/+,CD38dim (186)	B cells reduced (185), but no change in Bmem percentages (186)	N/A	-Inhibits B cell proliferation (187)	RRMS -Reduced annualized relapse rate -Fewer patients experience 3 month sustained disability worsening -More patients relapse free -Reduced MRI total lesion volume and gd-enhancing lesions	FDA approved: RRMS
Mitoxantrone	synthetic anthracenedione derivative	Immunosuppressive including B cell, T helper and T cytotoxic lymphocytes (188, 189)	CD19+, CD27+ (85)	Decreased (85)	N/A	No effect of B cell proliferation (188) -Preferential death of CD27+ B cells *vs* CD27- B cells B cells show decrease in lymphotoxin and TNF-α production (85) Increased IL-10 *in vitro* (85)	RRMS: -Reduced proportion of patients with confirmed progression over 2 years -prolongs time to first treated relapse SPMS: -delayed progression -Reduced new T2 lesions	FDA approved: RRMS SPMS
Alemtuzumab	Humanized mAb IgGk anti-CD52	-High levels on T and B cells -Lower levels on NK cells, monocytes, DCs, macrophages, and eosinophils -Relative sparing of Tregs and little/no expression on neutrophils, plasma cells, hematopoietic precursor cells (190, 191)	CD19+, CD27+ (192)	Decreased (192)	N/A	N/A	RRMS: -Reduced annualized relapse rate *vs* subcutaneous IFNβ-1a -Six-month sustained accumulation of disability reduced -Improvement of EDSS -Increased patients free from any clinical/MRI disease activity	FDA approved: RRMS
Natalizumab	humanized IgG1 mAb to α_4_β_1_ integrin	All leukocytes except neutrophils (193, 194)	CD19, +, CD27+, IgD+ (193) CD27var, CD38- (178)	Increased (178, 193)	Decreased B cell percentages (179); Bmem and plasmablasts (93)	- increased CD95+ B cells, increased MHCII+ B cells, increased CD40+ b cell percentage, and increases TNF and IL-6 in *in vitro* stimulated B cells (178)	RRMS: -Reduced annualized relapse rate Reduced risk of sustained disability worsening at 2 years -Decreased gd-enhancing lesions and new/enlarging T2-hypointense lesions	FDA approved: RRMS
Daclizumab	Humanized IgG1 mAb to CD25	Primarily CD4 T cells, but also activated CD8 T cells, dendritic cells, NK cells, and activated B cells and Bmem (195)	CD19+, CD27+	Decreased	N/A	N/A	RRMS: -Reduced annualized relapse rate -Reduced contrast-enhancing lesions and new/enlarging T2 lesions -Improved clinical rating scales	FDA approved: RRMS

ADCC, antibody-dependent cytotoxicity; CDC, complement-dependent cytotoxicity; CSF, cerebrospinal fluid; EDSS, Expanded Disability Status Scale; gd, gadolinium; mAb, monoclonal antibody; N/A, not available; PB, peripheral blood; PPMS, primary-progressive multiple sclerosis; RRMS, relapsing-remitting multiple sclerosis; SPMS, secondary progressive multiple sclerosis; var, variable.

Further B cell-directed therapies have sought to target a more diverse range of B cell phenotypes. Inebilizumab (MEDI-551), an anti-CD19 monoclonal antibody targets pro-B cells through memory B cells, plasmablasts, and some plasma cells (155, 198). In contrast to CD20 which is also expressed on a subpopulation of CD4^+^ T cells, CD19 is exclusively expressed on B cells (198). Similar to anti-CD20 directed therapies, treatment in RRMS patients results in reduced peripheral B cells (156, 157) and decreased gadolinium-enhancing lesions (157). B cell immunomodulatory therapies targeting B cell survival factors have shown contrasting effects on clinical outcomes. Atacicept treatment in RRMS patients resulted in an increased annualized relapse rate and unaltered gadolinium-enhancing lesions leading to the early termination of the phase II clinical trial (199). In rheumatoid arthritis patients, atacicept treatment led to an increase Bmem numbers in the peripheral blood (152), confirming previous studies that Bmem are spared (137). Similarly, tabalumab, an anti-BAFF monoclonal antibody which blocks immature B cells, mature B cells, and ASC survival, also fails to deplete Bmem (153, 154). Bmem were increased in the peripheral blood (154) and no reduction in gadolinium-enhancing lesions was observed in RRMS patients (200). The findings of unchanged or worse clinical outcomes in atacicept and tabalumab may be due to the minimal effect on Bmem (152, 201), although further studies are required.

Recently, the landscape of MS therapies targeting B cells has expanded to include Bruton’s tyrosine kinase (BTK) inhibitors. BTK is a critical enzyme for signaling through the BCR, FcγR, and GM-CSF receptor and is therefore involved in both adaptive and innate immune responses (160, 202). BTK inhibition affects myeloid cells, including microglia (203), and other hematopoietic lineage cells with exception to T cells, plasma cells, and natural killer cells (161). As small molecules, many BTK inhibitors also rapidly penetrate the blood-brain barrier (202, 203). The BTK inhibitors evobrutinib, tolebrutinib, fenebrutinib, orelabrutinib, and B11091 are currently in clinical development for relapsing and progressive forms of MS (**Table 1**). In clinical trials, BTK inhibitors were shown to reduce gadolinium-enhancing lesions (204) and new or enlarging T2 hypointense lesions (205), but did not reduce annualized relapse rates or disease progression in RRMS patients (204). Preliminary studies monitoring peripheral blood B cells in evobrutinib-treated RRMS and SPMS patients revealed no clinically relevant changes in the number of total B cells or Bmem over the 48 week treatment period (162). However, *in vitro* assays demonstrated an alteration in Bmem function, with reduced CXCR3+ Bmem migration across human brain endothelial cells (206).

### Other Immunomodulatory Therapies

Numerous immunomodulatory therapies utilized in MS have also been observed to affect Bmem. Although not traditionally viewed as modulating the B cell compartment, these therapies can have direct or indirect effects on Bmem survival and function. Interferon (IFN)-β, glatiramer acetate, fingolimod, dimethyl fumarate, and mitoxantrone all reduce Bmem numbers in peripheral blood and alter global B cell function following therapeutic treatment (**Table 1**). Peripheral blood B cells obtained from IFN-β-treated patients exhibit reductions in MHCII expression (167), reduced co-stimulatory molecules CD80 (168) and CD40 (169), and an increase in IL-10 production (168, 170), suggesting a shift in the overall B cell profile to an anti-inflammatory state. IFN-β treatment was also found to increase Bmem apoptosis (115). Glatiramer acetate-treated MS patients also show alterations in B cell function, resulting in reduced activation markers (CD69, CD95), decreased TNF production, and increased IL-10 production (173). Fingolimod, which targets SIP receptor-expressing lymphocytes such as T cells and B cells results in impaired CSF B cell clonal expansion (93), including Bmem, and reduced Bmem activation in peripheral blood from MS patients (177). Dimethyl fumarate treatment results in similar modulation reducing B cell activation (183) and the production of the pro-inflammatory cytokines GM-CSF, TNF, and IL-6 (181, 183), while IL-10 production is unaltered (182). Mitoxantrone treatment, immunosuppressive to T cells and B cells, does not affect B cell proliferation (188), but results in the preferential death of CD27-expressing B cells and a shift to an anti-inflammatory state, with reduced LT and TNF production, and increased IL-10 production *in vitro* (85). Conversely, natalizumab, which blocks leukocyte α_4_β_1-_mediated entry into the CNS, results in a 2.4-fold increase in Bmem in the peripheral blood (178, 193), but a reduction of Bmem in the CSF (93). In contrast to the aforementioned therapies, B cell activation (CD95, CD40, MHCII expression) and TNF and IL-6 production was increased in the peripheral blood of natalizumab-treated MS patients (178). Multiple other immunomodulatory therapies which have shown to be effective in improving clinical outcomes in RRMS patients, including cladribine, teriflunomide, daclizumab, and alemtuzumab all decrease peripheral Bmem numbers (**Table 1**), though findings related to the functional changes in B cells following therapeutic treatment remain to be determined.

### Bmem and Tailoring Therapeutic Treatment

Bmem in peripheral blood may prove useful for monitoring therapeutic effects in MS. In one study, Novi et al. utilized a Bmem-based reinfusion protocol for rituximab administration. Bmem monitoring (CD19^+^ CD27^+^ PBMCs) was used to orchestrate rituximab reinfusion, leading to a reduced number of reinfusions while still reducing disease activity (146). This study highlights the potential role for monitoring Bmem to tailor immunomodulatory treatments in MS. Future studies may also investigate the utility of monitoring Bmem in peripheral blood to predict response to therapy, including B cell depletion, in MS. Bmem monitoring in peripheral blood is a currently utilized strategy for predicting response to B cell depletion therapies in several autoimmune diseases implicating B cells including Sjogren’s syndrome, system lupus erythematosus, and rheumatoid arthritis (207–209).

Altogether, future studies are required to determine the exact effects on Bmem function following immunomodulatory treatment, including whether Bmem are central to the efficacy of disease-modifying therapies, and whether Bmem monitoring can be used to “personalize” immunotherapy.

## Concluding Remarks and Future Directions

**Figure 3:**
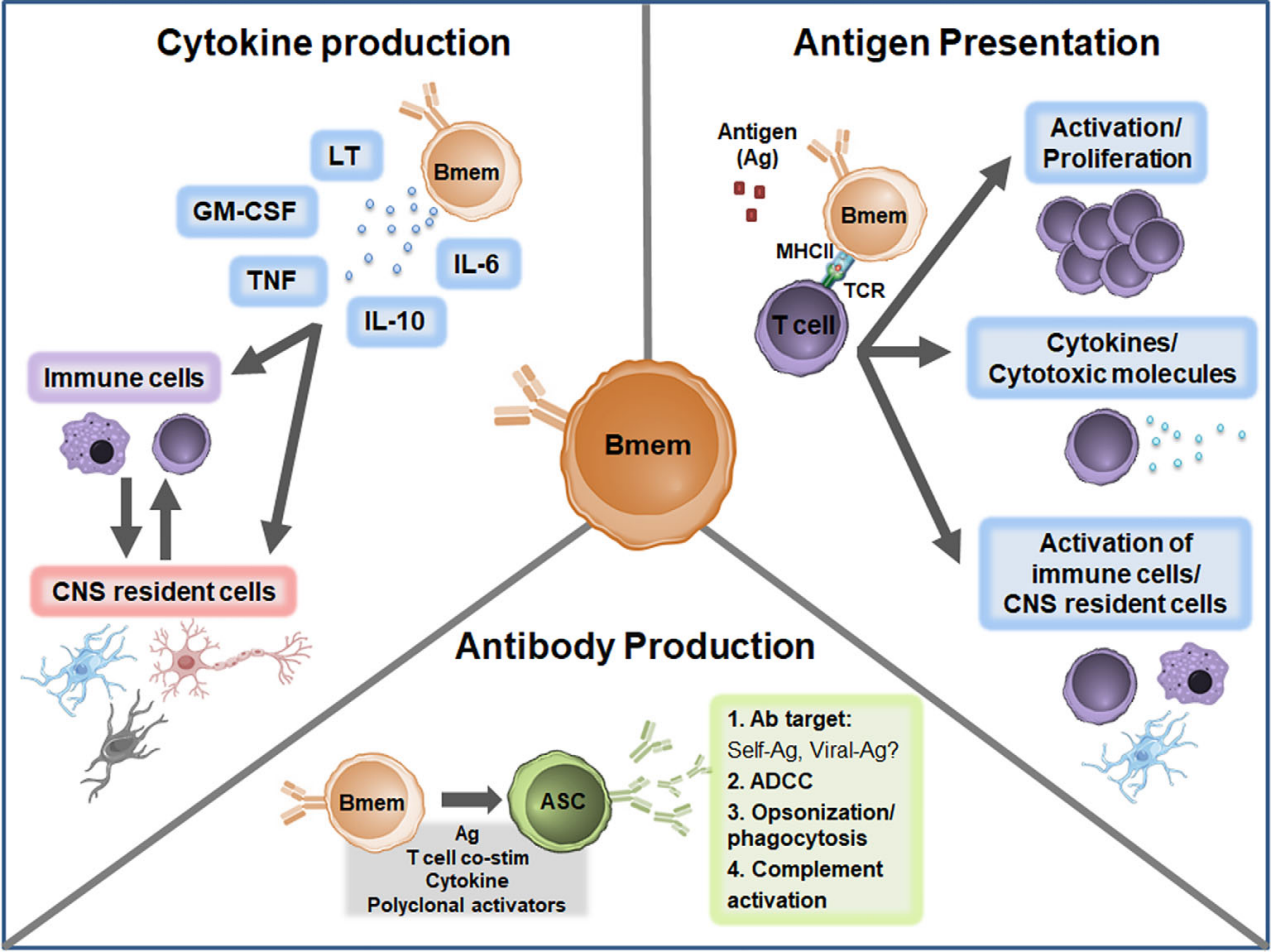
Proposed Bmem functions in MS. Canonical Bmem functions include cytokine production, antigen presentation, and antibody production. Bmem may produce a variety of pro-inflammatory and anti-inflammatory cytokines in MS, including lymphotoxin (LT), GM-CSF, TNF, IL-10, and IL-6. The production of these cytokines may 1) modulate the inflammatory function of immune cells, including monocytes and T cells in the periphery or CNS or 2) may alter the function and survival of CNS resident cells, including neurons, astrocytes, microglia, and oligodendrocytes. Bmem-derived cytokines may also modulate the interactions between CNS-localized immune cells and CNS resident cells. Bmem are potent antigen presenting cells (APCs) and upon uptake and presentation of antigen (Ag) may interact with other immune cells, including T cells, to enhance cell proliferation and effector functions. For example, following T cell-Bmem interaction, activated T cells may engage in cytokine production or cytotoxic molecule secretion. Bmem antigen presentation to T cells may also modify T cell engagement with other immune cells in the periphery or CNS and interaction with CNS resident cells. Bmem can differentiate into ASC following stimulation, including re-exposure to antigen, T cell co-stimulation (co-stim), cytokine stimulation, or T cell-independent polyclonal stimulation. Upon differentiation, Bmem-derived ASC may be involved in sustaining antibody responses in the CNS compartment. Bmem-derived ASC may also contribute to several antibody-dependent functions implicated in MS, including targeting self or viral antigens, antibody-dependent cellular cytotoxicity (ADCC), opsonization/phagocytosis, and complement engagement.

The cause of MS is unknown but growing evidence suggests multiple B cell phenotypes are central players in MS pathogenesis. In MS, Bmem in both the peripheral and CNS compartments are increasingly being explored to define the exact relationship with disease development and progression. Important observations highlighted in the current review include the presence of Bmem alterations in both the peripheral blood and CNS compartments in MS; evidence for potential roles in antibody production, antigen presentation, and cytokine production (**Figure 3**); and effective targeting of Bmem using currently available immunomodulatory therapies. Future studies should aim to address several key unresolved questions to provide more in-depth insights regarding Bmem in MS (**Table 2**), including trafficking mechanisms, action within the CNS compartment, functional relevance in MS immunopathogenesis, and defining associations with clinical outcomes. These insights may help to guide therapeutic strategies to develop novel agents specific for Bmem and tailor current therapeutic treatment regimens.

**Table 2:** Bmem in MS: Unresolved questions.

What mechanisms promote Bmem trafficking to the CNS? Adhesion molecules, chemokines etc.
Do Bmem participate in meningeal inflammation?
Do Bmem play a significant role in sustaining local ASC/Ab in the CNS?
What is the antigen specificity of Bmem recruited to the CNS? Is it the antigen diversity similar to CSF Abs?
Are Bmem pro-inflammatory, anti-inflammatory, or do Bmem play a pleiotropic role in MS?
Do Bmem phenotypes, kinetics, and functions differ by MS disease phenotype?
How do Bmem interact with other immune cells and CNS resident cells within the CNS?
How can Bmem be utilized to monitor and optimize therapeutic effects?

## Author Contributions

KD and AP outlined the subject for the review. KD reviewed the literature, drafted the figures and tables, and wrote the manuscript. AP and FG edited and revised the manuscript. All authors contributed to the article and approved the submitted version.

## Funding

Dartmouth College’s Open-Access Publication Equity Fund sponsored by the Dartmouth Library and Office of the Provost.

## Conflict of Interest

The authors declare that the research was conducted in the absence of any commercial or financial relationships that could be construed as a potential conflict of interest.
